# Roles of IL-22 in Allergic Airway Inflammation

**DOI:** 10.1155/2013/260518

**Published:** 2013-02-21

**Authors:** Koichi Hirose, Kentaro Takahashi, Hiroshi Nakajima

**Affiliations:** Department of Allergy and Clinical Immunology, Graduate School of Medicine, Chiba University, 1-8-1 Inohana, Chiba City, Chiba 260-8670, Japan

## Abstract

IL-23- and IL-17A-producing CD4^+^ T cell (Th17 cell) axis plays a crucial role in the development of chronic inflammatory diseases. In addition, it has been demonstrated that Th17 cells and their cytokines such as IL-17A and IL-17F are involved in the pathogenesis of severe asthma. Recently, IL-22, an IL-10 family cytokine that is produced by Th17 cells, has been shown to be expressed at the site of allergic airway inflammation and to inhibit allergic inflammation in mice. In addition to Th17 cells, innate lymphoid cells also produce IL-22 in response to allergen challenge. Functional IL-22 receptor complex is expressed on lung epithelial cells, and IL-22 inhibits cytokine and chemokine production from lung epithelial cells. In this paper, we summarize the recent progress on the roles of IL-22 in the regulation of allergic airway inflammation and discuss its therapeutic potential in asthma.

## 1. Introduction

 Asthma is a chronic inflammatory disease that is accompanied by intense eosinophilic infiltration, goblet cell hyperplasia, and airway hyperreactivity (AHR) [1]. In atopic asthma patients, it is well established that these features are mediated by antigen-specific Th2 cells and their cytokines including IL-4, IL-5, and IL-13 [2, 3]. In addition, several lines of evidence have shown that not only Th2 cell-derived cytokines but also Th17 cell-derived cytokines such as IL-17A and IL-17F are expressed in the airways in severe asthma patients, and that the levels of IL-17A and IL-17F in the airways are correlated with the severity of asthma, suggesting the involvement of Th17 cell-derived cytokines in the pathogenesis of severe asthma [4, 5]. Moreover, we and others have shown that Th17 cells are involved in the development of antigen-induced airway inflammation in murine asthma models [6–8]. Interestingly, recent studies have shown that IL-22, one of Th17 cell-derived cytokines, is detected in bronchoalveolar lavage fluid (BALF) in murine asthma models [8, 9]. Furthermore, it has been reported that the levels of IL-22 mRNA are increased in peripheral blood mononuclear cells in asthma patients [10, 11], and that the levels of IL-22 in sera tend to correlate with the severity of asthma [12]. In this paper, we briefly summarize the roles of IL-22 in the regulation of allergic inflammation in asthma.

## 2. IL-22 and IL-22 Receptor

 IL-22 is an IL-10 family cytokine that is originally identified from IL-9-stimulated T lymphoma cells and designated as IL-TIF (IL-10-related T cell-derived inducible factor) [13]. Functional IL-22 receptor consists of IL-22R1 and IL-10R2, which are associated with tyrosine kinases Jak1 and Tyk2, respectively [14–16]. IL-22R ligation activates not only STAT pathways (STAT1, STAT3, and STAT5), but also JNK, ERK, and p38 MAP kinase pathways [16]. In spite of the wide range activation of signaling pathways, however, STAT3-mediated signaling seems to be the major pathway in IL-22 signaling [17]. 

 Interestingly, IL-22 possesses both proinflammatory and anti-inflammatory properties depending on environmental context. While a number of studies have shown that IL-22 plays protective roles in host defense against infectious diseases, some studies have shown that IL-22 is involved in the development of autoimmune diseases [18]. In IL-23-induced dermatitis in mice, IL-22 is crucial for the induction of dermal inflammation and acanthosis [19]. On the other hand, IL-22 plays a protective role in hepatitis and inflammatory bowel disease models [20, 21]. As described below, we and others have found that IL-22 attenuates antigen-induced allergic inflammation and Th2 cytokine production in the airways in mice presumably through the inhibition of cytokine and chemokine production from lung epithelial cells [22–24]. 

## 3. Roles of IL-22 in Murine Asthma Models 

 Recently, we and others have explored the roles of IL-22 in the pathogenesis of allergic airway inflammation and have shown that IL-22 expression is induced in the lung of antigen-sensitized mice upon antigen inhalation [8, 9, 22–24]. In addition, the administration of a neutralizing anti-IL-22 antibody significantly enhances antigen-induced infiltration of eosinophils, Th2 cytokine production in the airways, and AHR in mice [9, 22, 24]. On the other hand, intranasal administration of recombinant IL-22 attenuates antigen-induced eosinophil infiltration into the airways, even if IL-22 is administered into the airways after the induction of allergic airway inflammation [22, 24]. Moreover, it has been demonstrated that enforced expression of IL-22 by gene delivery suppresses eosinophilic airway inflammation and Th2 cytokine production in the airways in mice [25]. Consistent with these findings, Taube et al. have reported that antigen-induced allergic inflammation is enhanced in IL-22-deficient mice [23]. These findings suggest that IL-22 has a protective role for the development of antigen-induced airway inflammation in mice during the effector phase. On the other hand, Besnard et al. have shown that IL-22 also plays a crucial role in antigen sensitization in a murine asthma model in which mice were sensitized with antigens subcutaneously [24]. Considering the contribution of percutaneous priming for the development of atopic march, a progression from eczema to allergic rhinitis and asthma [26, 27], their finding may suggest that IL-22 plays a role in percutaneous sensitization and thus the development of asthma in patients with eczema. Taken together, based on these murine studies, we propose that IL-22 has a double-edged nature in allergic airway inflammation, depending on the timing or the site of its expression.

 IL-22-binding protein (IL-22BP), which is highly homologous to the extracellular domain of IL-22 receptor, is considered as an endogenous antagonist for IL-22 [28]. Interestingly, IL-22BP is highly expressed in lung and colon, where IL-22 exhibits a regulatory role [28]. Moreover, by using IL-22BP-deficient mice, Huber et al. have recently demonstrated that the downregulation of IL-22BP, thereby increasing the ratio of IL-22/IL-22BP, is induced during intestinal tissue damage and thus contributes to the protective properties of IL-22 [29], suggesting that IL-22BP functions as an intrinsic inhibitor of IL-22 in the colon. However, it remains unknown whether IL-22BP plays a role in allergic airway inflammation in mice and whether IL-22BP has equivalent properties in humans.

## 4. Cellular Sources of IL-22 in the Lung in Murine Asthma Models

 It has been reported that not only CD4^+^ T cells including Th17 cells and Th22 cells, but also some populations of innate immune cells including NK cells, dendritic cells (DCs), and lymphoid tissue inducer-like cells (LTi-like cells) are capable of producing IL-22 [30–35]. We have shown that the majority of IL-22-producing cells at the site of allergic airway inflammation are CD4^+^ T cells and that one third of the IL-22-producing CD4^+^ T cells produce IL-17A [22], suggesting that some of IL-22-producing CD4^+^ T cells in a murine asthma model are Th17 cells. We have also shown that NK cells, DCs, and LTi-like cells do not express IL-22 mRNA in the lung in the murine asthma model [22]. In contrast, Taube et al. have reported that ROR*γ*t-expressing LTi-like cells are the major cellular sources of IL-22 in the lung in a different murine model of asthma [23]. In this regard, a recent study in a murine intestinal infection model has shown that IL-22 produced by CD4^+^ T cells contributes to late-phase responses to an intestinal pathogen, while IL-22 produced by innate cells plays a critical role in early-phase responses [35]. In analogy to these findings, IL-22 produced by LTi-like cells and that produced by CD4^+^ T cells may play a distinct role in allergic airway inflammation depending on the phase of responses.

## 5. Mechanisms Underlying IL-22-Mediated Inhibition of Allergic Airway Inflammation

 The functional IL-22 receptor is a heterodimer of IL-22R1 and IL-10R2 and previous studies have shown that whereas IL-10R2 is ubiquitously expressed in various cells, the expression of IL-22R1 is restricted to nonimmune cells [14, 15]. Indeed, in a murine model of asthma, IL-22R1 is expressed in lung epithelial cells, but not in hematopoietic cells in the lung including alveolar macrophages, CD4^+^ T cells, and CD8^+^ T cells [22]. While a previous report suggested that DCs mediate the inhibitory function of IL-22 in mice [9], we failed to detect IL-22R1 mRNA expression in bone marrow-derived DCs [22]. Moreover, Nakagome et al. have reported that enforced expression of IL-22 does not affect DC functions in mice [25], supporting the notion that DCs are not direct targets of IL-22. Our finding that IL-22 phosphorylates STAT3, a signal transducer of IL-22, in lung epithelial cell line [22] further suggests that functional IL-22 receptor complex is expressed on lung epithelial cells and that direct targets of IL-22 in a murine model of asthma are lung epithelial cells. 

 Recently, there has been great progress in understanding the mechanism by which lung epithelial cells regulate the development of allergic inflammation [36]. Lung epithelial cells have been shown to produce several cytokines which promote Th2 responses and chemokines which attract immune cells into the lung in both mice and humans [36]. Importantly, anti-IL-22 antibody treatment significantly enhances the expression of IL-25, one of the epithelial cell-derived cytokines which promote Th2 responses, in the BALF in a murine model of asthma [22]. In addition, it has been shown that anti-IL-22 antibody enhances the production of IL-33, which also promotes Th2 responses, in different murine models of asthma [24, 37]. Furthermore, IL-22 has been shown to inhibit the expression of CCL17, which induces the recruitment of activated T cells into the lung, in a murine Clara cell line [23] as well as in a murine model of asthma [24]. These findings suggest that IL-22 may inhibit antigen-induced airway inflammation by suppressing cytokine and chemokine production from lung epithelial cells.

 The fact that IL-22 enhances the expression of host-defense peptides in epithelial cells is now widely accepted. In addition, it has recently been demonstrated that IL-22 enhances mucosal barrier function by protecting intestinal stem cells during inflammatory intestinal damage [38]. Several lines of evidence have suggested that the disruption of barrier function of lung is associated with the development of allergic airway inflammation by facilitating the entry of allergens into the tissue [39, 40]. These findings raise the possibility that IL-22 may suppress the development of allergic airway inflammation by enhancing the barrier function of airway epithelial cells. 

## 6. IL-22 and Asthmatic Patients 

 It has been reported that IL-22 levels are increased in the sera of asthma patients and are positively correlated with disease severity [11, 12, 24]. The majority of IL-22-producing cells in peripheral blood of asthma patients are CD4^+^CCR6^+^CD161^+^ cells [41], suggesting that Th17 cells are the main producer of IL-22. In contrast, it has been reported that the majority of IL-22-producing CD4^+^ T cell lines which are generated from lung biopsy specimens of asthma patients produce IFN-*γ* [42]. The reason for the discrepancy is at present unclear and further studies to verify the cellular source of IL-22 in asthma patients are required.

 Regarding the function of IL-22 in asthma patients, Pennino et al. have recently shown that IL-22 inhibits IFN-*γ*-induced expression of proinflammatory chemokines and adhesion molecules in human bronchial epithelial cells [42]. They have also shown that the levels of IL-22 in the BALF of asthma patients are inversely correlated with the levels of proinflammatory chemokines, suggesting the protective roles of IL-22 in asthma patients [42]. On the other hand, it has been shown that IL-22R1 is expressed on not only lung epithelial cells but also airway smooth muscle cells (ASMCs) in humans and that IL-22 enhances the proliferation and migration of human ASMCs [43–45], suggesting that IL-22 may involve in smooth muscle cell hyperplasia, a key pathological feature of asthma, in human airways. Taken together, these human studies suggest that IL-22 plays inhibitory roles in the development of allergic airway inflammation in asthma patients, but it could promote airway remodeling if its expression is uncontrolled during the resolution phase of allergic inflammation. 

## 7. Concluding Remarks

**Figure 1:**
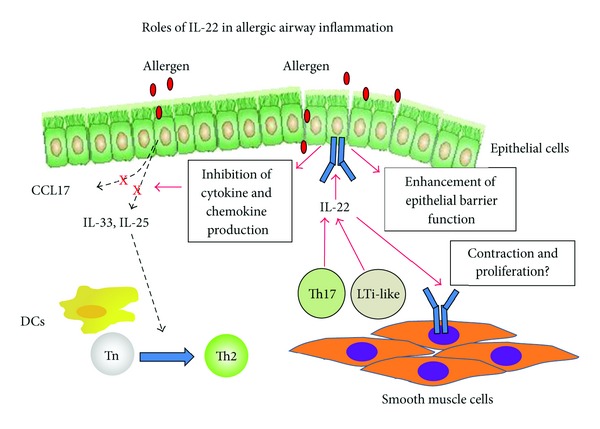
Roles of IL-22 in allergic airway inflammation. Upon antigen inhalation, CD4^+^ T cells and LTi-like cells in the lung produce IL-22. IL-22 inhibits the expression of lung epithelial cell-derived cytokines and chemokines, including IL-25, IL-33, and CCL17, and attenuates the development of allergic airway inflammation. In addition, IL-22 may enhance barrier function of airway epithelial cells, which may contribute to the protective function of IL-22 in asthma. On the other hand, IL-22 may cause the proliferation of airway smooth muscle cells in humans, which may lead to airway remodeling in asthma.

 In this paper, we outlined the roles of IL-22 in the development of allergic airway inflammation. Based on the findings of murine asthma models, IL-22 is produced by Th17 cells and LTi-like cells at the site of allergic airway inflammation and attenuates eosinophilic inflammation and AHR, presumably by inhibiting cytokine and chemokine production from lung epithelial cells (Figure 1). In asthma patients, however, excessive production of IL-22 may lead to the progression of airway remodeling by enhancing the proliferation and migration of ASMCs. Further studies to elucidate the precise function of IL-22 at different stages of asthma pathogenesis will provide a great benefit for the development of a novel therapeutic approach for asthma.
